# Untypical Amlodipine-Induced Gingival Hyperplasia

**DOI:** 10.1155/2015/756976

**Published:** 2015-01-27

**Authors:** Verica Pavlic, Nina Zubovic, Sanja Ilic, Tijana Adamovic

**Affiliations:** ^1^Department of Periodontology and Oral Medicine, Institute of Dentistry, Zdrave Korde 4, 78000 Banja Luka, Bosnia and Herzegovina; ^2^Department of Endodontics, Institute of Dentistry, Zdrave Korde 4, 78000 Banja Luka, Bosnia and Herzegovina; ^3^Department of Periodontology and Oral Medicine, Medical Faculty University of Banja Luka, Bulevar Petra Bojovica 1, 78000 Banja Luka, Bosnia and Herzegovina

## Abstract

Amlodipine is a third generation dihydropyridine calcium channel blocker that is frequently used in therapy of hypertension. Among many side effects, amlodipine has been found associated with gingival overgrowth (GO) which usually occurs within the first three months of starting therapy at a dose of 10 mg/day. However, there are very few reports on amlodipine-induced gingival overgrowth (AIGO) at a lower dose (5 mg/day) and only after short term administration. A 64-year-old male patient with hypertension, who received amlodipine (5 mg/day) for four years, sought medical attention at the Department of Periodontology and Oral Medicine, Institute of Dentistry, Banja Luka, Bosnia and Herzegovina. The patient complained of masticatory problems due to extensive maxillary GO along with pain, bleeding, and foul odor. The clinical and the histological evidences were consistent with AIGO. The first line treatment consisted of the amlodipine substitution (amlodipine was replaced with enalapril, 5 mg/day) and the scaling and root planning/SRP. At one-month follow-up, drug replacement and SRP resulted in some reduction of the inflammation and significant reduction of symptoms. Further, gingivectomy/gingivoplasty helped overcome the effect of these drugs. The possibility of AIGO should be considered for a lower dose, as well as a late presentation.

## 1. Introduction

Gingival overgrowth (enlargement, hyperplasia) is benign painless condition, characterized by massive enlargement of the interdental papillae, which can range from mild to extremely severe [1–3]. It may be accompanied by swelling of the gingival margin and partial cover of the occlusal surface of teeth, causing aesthetic limitations and functional difficulties in swallowing, speaking, and mastication [1, 2]. Gingival overgrowth (GO) is usually more conspicuous at labial/buccal surface of both upper and lower anterior teeth [4]. GO can be caused by various factors, such as inflammatory changes, mouth breathing, vitamin C deficiency, heredity, malignancies, hormonal alterations (seen in puberty and pregnancy), and the adverse effects associated with the systemic administration of certain drugs/drug-induced gingival overgrowth (DIGO). The prevalence of the DIGO is 3–20% compared to other gingival enlargements [4]. DIGO was firstly observed in patients who were taking phenytoin for epilepsy, with approximately 50% having GO [1–6]. Currently, more than 20 drugs, mainly among anticonvulsants (e.g., phenytoin), immunosuppressants (e.g., cyclosporine A), and various calcium channel blockers (e.g., nifedipine, verapamil, and diltiazem), are associated with gingival enlargements [3–7]. The pharmacologic effect of each of these drugs is different but all of them seem to act similarly on secondary target tissue, such as gingival tissue, resulting in similar clinical and histopathological findings. Among calcium channel blockers, the dihydropyridines (e.g., nifedipine, felodipine, amlodipine, nitrendipine, nicardipine, and manidipine) tend to be more commonly associated with gingival enlargement [7]. Amlodipine is a third generation dihydropyridine calcium channel blocker that is frequently used in therapy of hypertension and angina pectoris [1]. The prevalence of GO associated with amlodipine is reported to be 3.3%, which is significantly lower than that associated with nifedipine, ranging from 14 to 83% [8].

Since the exact pathogenesis of amlodipine-induced gingival hyperplasia (AIGO) is not well-understood, it has become a serious challenge for both the patients and dentists/periodontists to diagnose and manage the cases effectively. The first line management of AIGO is withdrawal or substitution of amlodipine with the patient's physician consent [1–4]. Unfortunately, in most of the cases, withdrawal is not possible and drug substitution alone is not enough to overcome the AIGO effects. Usually GO are traps for debris and plaque, causing difficulties in maintenance of oral hygiene, further enhancing secondary inflammation and susceptibility of periodontal disease and caries [1–3]. Therefore, stringent maintenance of oral hygiene measures, ensuring that the accumulated plaque is removed from around the necks of the teeth and gums (professional tooth cleaning, scaling, and root planning/SRP), is, however, necessary [1–4, 9]. Situations when GO persists after careful consideration of the previously mentioned approaches and/or in which the chronic inflammatory gingival enlargement includes significant fibrotic components are treated with surgical removal of the excess tissue, either gingivectomy/gingivoplasty or flap surgery [9, 10].

To date reports related to AIGO are very rare. Majority of available literature are case studies/presentations which demonstrated that the AIGO occurs within 2-3 months of onset at a dose of 10 mg/day and rarely within first 6 months of onset at a lower dose of 5 mg/day [3]. The present case is interesting as AIGO occurred with a low dose of amlodipine (5 mg/day) and appeared on after several years of administration.

## 2. Case Report

**Figure 1:**
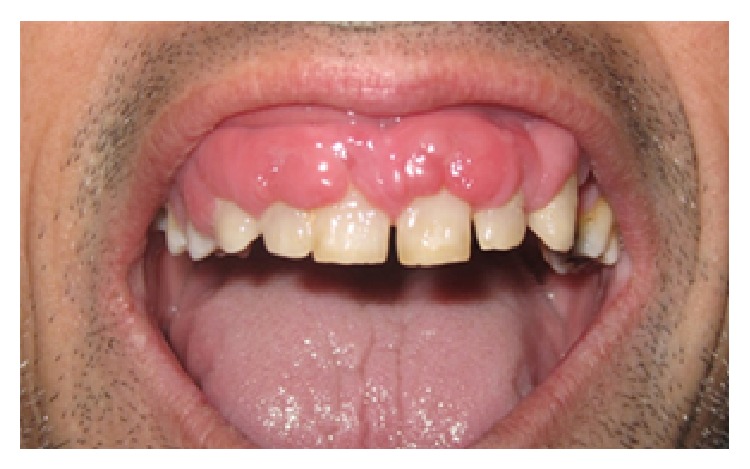
Clinical presentation of generalized diffused gingival overgrowth of upper arch (at patient's first visit).

**Figure 2:**
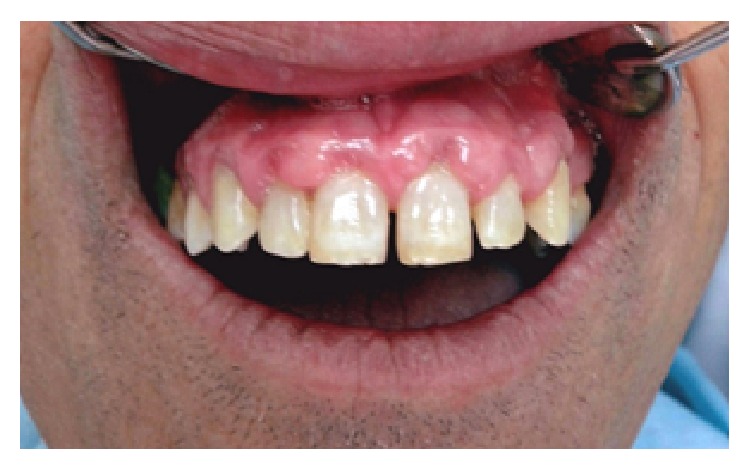
Gingival overgrowth one month after therapy (visible regression after amlodipine substitution and SRP).

A 64-year-old male patient with hypertension, who received amlodipine (Amlopin, Lek, Ljubljana, Slovenia) 5 mg/day, one dose orally for four years, sought medical attention at the Department of Periodontology and Oral Medicine, Institute of Dentistry, Banja Luka, Bosnia and Herzegovina, with the chief complaint of the swollen gums. Until 3 months back, patient firstly noted bead like nodular growth over the upper arch gums which progressively enlarged causing masticatory problems due to extensive gingival overgrowth along with pain, bleeding, and foul odor. The medical history of the patient revealed that the patient was hypertensive and that he was under amlodipine for a period of 4 years. The physical examination revealed noncyanosed moderately built and nourished man with no signs of anaemia. His vital signs were within the normal range. An intraoral examination revealed generalized diffused GO in the maxilla. The enlarged gingiva was firm, pink, and resilient with tendency to bleed (Figure 1). Poor oral hygiene status of patient was assessed by the presence of local irritating factors which surrounded the teeth. Due to the outward (rather than vertical) enlargement of gingiva, the deep periodontal pockets were not detected. Orthopantomogram revealed generalized moderate horizontal bone loss. Based on drug history and clinical examination of the patient, provisional diagnosis of combined GO (amlodipine-induced GO complicated by inflammatory effects) was made. After this, incisional gingival biopsy was done. Histopathological report revealed the increase in the gingival connective tissue volume (a mixture of dense and loose fibrous components) with the chronic inflammatory cell infiltrate and acanthosis of overlying epithelium. According to medical history, clinical examination, and investigations, final diagnosis of AIGO was made. Firstly, a patient's physician was consulted in order to substitute amlodipine, whom replaced amlodipine with angiotensin-converting-enzyme (ACE) inhibitor enalapril (Enap, Krka, Novo Mesto, Slovenia) 5 mg/day, one dose orally. Along with drug substitution, complete SRP was performed for supragingival and subgingival calculus removal. At subsequent follow-up after one month, first line therapy resulted in some reduction of the inflammatory component, reduction of the size of the GO, and significant reduction of symptoms (Figure 2). Later, remaining aesthetic and functional limitations were overcome by the surgical reduction of the enlarged tissue (gingivectomy and gingivoplasty).

## 3. Discussion

AIGO has a multifactorial nature and its appearance and severity are strongly influenced by dosage, duration, and blood level of amlodipine, as well as sex, genetic predisposition (fibroblasts with an abnormal susceptibility to the drug and/or the functional fibroblast heterogeneity), oral hygiene status/preexisting gingival inflammation, and activation of growth factors [3, 4, 9]. The underlying mechanism behind AIGO is still not completely understood [3, 4]. It is basically described as multifactorial model, involving noninflammatory and inflammatory mechanisms [3, 4]. The proposed noninflammatory mechanisms include defective collagenase activity due to decreased levels of matrix metalloproteinases-1 and -3 secretions and uptake of folic acid, blockage of aldosterone synthesis in zona glomerulosa of the adrenal cortex, and upregulation of keratinocyte growth factor [1]. However, majority of the available literature suggested that the gingival inflammation is essential for the interaction between drugs and fibroblasts [11]. An inflammation may develop as a result of direct toxic effects of concentrated drug in crevicular gingival fluid, since the crevicular fluid concentrations of the drugs were found to be up to 292 times of those found in plasma [9]. This inflammation could lead to the upregulation of several cytokine factors such as fibroblast growth factor-2 (FGF-2), transforming growth factor-*β*1 (TGF-*β*1), interleukin-6 and interleukin-1*β* (IL-6, IL-1*β*), and platelet derived growth factor-*β* (PDGF-*β*) predisposing the tissue to a localized toxic effect and the development of fibrotic gingival hyperplasia [5]. Released proinflammatory cytokines are also involved in the mast cells migration, influencing fibroblast proliferation, extracellular matrix synthesis, and degradation. Further, amlodipine may stimulate the production of IL-2 by T-cells causing fibrosis [9]. The strong relation between the inflammation and AIGO is demonstrated with the fact that AIGO can be successfully controlled even under the continuous amlodipine administration by meticulous professional and individual oral hygiene [8–10]. Also, the fact that DIGO, in general, does not affect edentulous areas further supported the statement that the presence of GO is most probably related to inflammatory factors from dentition, such as dental plaque [8]. However, the evidence based clinical trials regarding the correlation between periodontal inflammation and AIGO are lacking.

Clinical manifestation of GO commonly appears within 2-3 months after initiation of treatment with the amlodipine, usually at a dose of 10 mg/day. There are very few reports on AIGO at a lower dose (5 mg/day) and only after short term administration. However, according to some authors, amlodipine dose of 5 mg/day cannot induce gingival hyperplasia even if taken more than 6 months [12]. In the present case, GO occurred with a low dose of amlodipine (5 mg/day) and appeared after several years of administration. It is still unknown why AIGO in this case exacerbates after 4 years of continuous use. Apart from previously mentioned well-known possible risk factors, our patient did not have a habit of regular/daily tooth brushing. Further, possible explanation is the fact that our patient was a man, and AIGO occurs three times more often in men than in women [13]. Another potential explanation could be a patient's sensitivity to a specific drug's metabolic pathway [13] or patient's sensitivity to a specific amlodipine concentration in the crevicular gingival fluid [7].

Medical doctors and dentists/periodontists should be aware of the use of medications with the potential to cause/contribute to the development of the GO, to prevent, diagnose, and successfully manage unwanted side effects of drugs by cooperative teamwork. Since it is expected that number of these drugs is likely to increase in the years to come, the thorough understanding and clear establishment of the DIGO molecular pathogenesis and novel preventive and treatment modalities are however necessary.

## 4. Conclusion

The gingival overgrowth could occur with amlodipine even at a small dose (5 mg/day) and after a long term application. Medical doctors and dental clinicians/periodontists need to be aware of the etiologic medications, such as amlodipine, that can induce GO in order to identify changes in the oral cavity in such patients and to prevent, diagnose, and successfully manage its unwanted side effects. The cooperative teamwork between the patient, his physician, and the periodontist is however necessary in order to obtain the best AIGO treatment outcome.
